# Di-μ-iodido-bis­{[(*R*)-(+)-2,2′-bis­(di­phenyl­phosphan­yl)-1,1′-binaphthyl-κ^2^ *P*,*P*′]copper(I)} 0.67-hydrate

**DOI:** 10.1107/S1600536812011051

**Published:** 2012-03-24

**Authors:** Daniel Volz, Martin Nieger, Stefan Bräse

**Affiliations:** a Institute of Organic Chemistry, Karlsruhe Institute of Technology (KIT), Fritz-Haber-Weg 6, 76128 Karlsruhe, Germany; b Department of Chemistry, University of Helsinki, PO Box 55 (A.I. Virtasen aukio 1), 00014 Helsinki, Finland

## Abstract

The structure of the title compound, [Cu_2_I_2_(C_44_H_32_P_2_)_2_]·0.67H_2_O, has been determined because of its inter­esting catalytic and optical features. The mol­ecule, which has non-crystallographic *C*2-symmetry, consists of a core structure of two Cu^I^ ions, bridged by two iodide ions. Each Cu^I^ ion is also coordinated by one equivalent of the chiral bidentate (*R*)-BINAP ligand [BINAP = 2,2′-bis­(diphenyl­phosphan­yl)-1,1′-binaphth­yl]. Thus, both cations show a distorted tetra­hedral geometry being surrounded by two I atoms and two P atoms from the (*R*)-BINAP ligands. The complex consists of isolated butterfly-shaped mol­ecules featuring an angle of 146.11 (2)° between adjacent CuI_2_ planes. The structure displays intra­molecular C—H⋯I hydrogen bonding and contains disordered water. The absolute configuration of this chiral complex was determined by anomalous dispersion effects.

## Related literature

For the photophysical properties of the title compound, see: Kunkely *et al.* (2008 ▶) and of analogous complexes see: Balamurugan *et al.* (2001 ▶); Hashimoto *et al.* (2011 ▶); Hattori *et al.* (2010 ▶); Lipshutz *et al.* (2004 ▶); Miyashita *et al.* (1980 ▶); Yersin *et al.* (2011 ▶); Zink *et al.* (2011 ▶).
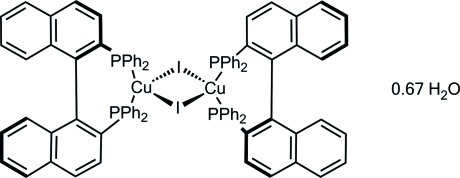


## Experimental

### 

#### Crystal data

[Cu_2_I_2_(C_44_H_32_P_2_)_2_]·0.67H_2_O
*M* *_r_* = 1638.16
Hexagonal, 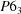

*a* = 25.573 (3) Å
*c* = 18.593 (2) Å
*V* = 10530 (2) Å^3^
*Z* = 6
Mo *K*α radiation
μ = 1.63 mm^−1^
*T* = 123 K
0.40 × 0.20 × 0.15 mm

#### Data collection

Bruker–Nonius KappaCCD diffractometer
Absorption correction: multi-scan (*SADABS*; Sheldrick, 1996 ▶) *T*_min_ = 0.696, *T*_max_ = 0.801
187180 measured reflections
16098 independent reflections
15085 reflections with *I* > 2σ(*I*)
*R*_int_ = 0.041

#### Refinement

*R*[*F* ^2^ > 2σ(*F* ^2^)] = 0.030
*wR*(*F* ^2^) = 0.081
*S* = 1.09
16098 reflections
873 parameters
1 restraint
H-atom parameters constrained
Δρ_max_ = 1.62 e Å^−3^
Δρ_min_ = −0.57 e Å^−3^
Absolute structure: Flack (1983 ▶), 7802 Friedel pairs
Flack parameter: −0.014 (9)

### 

Data collection: *COLLECT* (Nonius, 1998 ▶); cell refinement: *EVALCCD* (Duisenberg *et al.*, 2003 ▶); data reduction: *EVALCCD*; program(s) used to solve structure: *SHELXS97* (Sheldrick, 2008 ▶); program(s) used to refine structure: *SHELXL97* (Sheldrick, 2008 ▶); molecular graphics: *SHELXTL-Plus* (Sheldrick, 2008 ▶); software used to prepare material for publication: *SHELXL97*.

## Supplementary Material

Crystal structure: contains datablock(s) I, global. DOI: 10.1107/S1600536812011051/bt5845sup1.cif

Supplementary material file. DOI: 10.1107/S1600536812011051/bt5845Isup2.mol

Structure factors: contains datablock(s) I. DOI: 10.1107/S1600536812011051/bt5845Isup3.hkl

Supplementary material file. DOI: 10.1107/S1600536812011051/bt5845Isup4.mol

Additional supplementary materials:  crystallographic information; 3D view; checkCIF report

## Figures and Tables

**Table 1:** Hydrogen-bond geometry (Å, °)

*D*—H⋯*A*	*D*—H	H⋯*A*	*D*⋯*A*	*D*—H⋯*A*
C40—H40⋯I1	0.95	3.02	3.894 (4)	153

**Table 2:** Comparison of selected bond distances and angles (Å, °) for two (*R*)-BINAP–Cu–halide complexes.

Halide *X*	Cu—*X*	Cu—P	*X*—Cu—*X*	*X*—Cu—P	P—Cu—P	Cu—*X*—Cu
Iodide	2.641	2.28	102.5	113.6	99.5	73.3
Chloride	2.378	2.260	98.0	114.9	100.2	81.3

Values for the iodide complex are from this work, while data for the chloride complex were taken from Hattori *et al.* (2010 ▶).

**Fig. 1.**
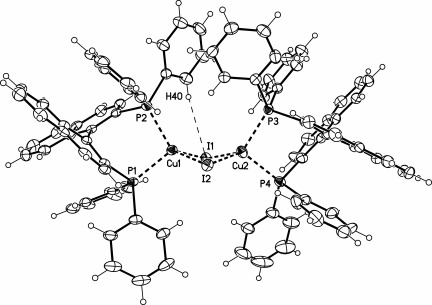
ORTEP-drawing of the title compound showing the coordination geometry of the butterfly-shaped Cu2I2-dimer and displacement ellipsoids of all non-H-atoms. (50% probability).

**Fig. 2.**
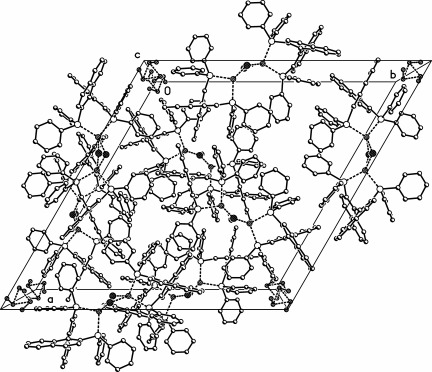
Packing diagram along the crystallographic c axis (hydrogen atoms omitted for clarity).

## supplementary crystallographic information

### Comment

The chiral arylphosphine 2,2'-bis(diphenylphosphanyl)-1,1'-binaphthyl, BINAP, has been introduced by Noyori and coworkers as a ligand suitable for rhodium(I)-catalyzed reductions of alpha-(acylamino)acrylic acids (Miyashita *et al.*, 1980). Various complexes analogous to the title compound are known: Copper(I)-complexes of arylphosphines such as BINAP have been studied *e.g.* as catalysts for an enantioselective amination-reactions with propargylic esters (Hattori *et al.*, 2010). Also, it has been demonstrated that (*R*)-BINAP can be removed from solutions by precipitation with CuCl as a 1:1 adduct, *e.g.* in order to retrieve chiral ligands after Pd-catalyzed cross coupling protocols (Lipshutz *et al.*, 2004). Hattori *et al.* determined the structure of the dimeric complex [(*R*)-BINAP(CuCl)]_2_ (Hattori *et al.*,2010), consisting of a butterfly-shaped Cu_2_I_2_-unit with one chelating BINAP-ligand coordinating each Cu^I^. However, the complexes of arylphosphanes and Cu^I^ have recently been studied due to their interesting photophysical properties (Zink *et al.*, 2011; Yersin *et al.*, 2011).

Vogler and coworkers analyzed the spectroscopic properties of a 1:1 adduct of BINAP and CuI, which proved to emit light at 582 nm even in solution (Kunkely *et al.*, 2008). The authors of that study suggested a structure comparable to [(*R*)-BINAP(CuCl)]_2_ for this complex, yet failing to provide any direct experimental proof for this thesis. Of course, the tetrahedral coordination geometry is dominant for copper(I) compounds, but some cases with a trigonal coordination have been found, mostly as a result of a complexation with bulky ligands (Hashimoto *et al.*, 2011; Balamurugan *et al.*, 2001).

Herein, we show that [(*R*)-BINAP (CuI)] is indeed a dimer (non-crystallographic C2-symmetry), very much comparable to [(*R*)-BINAP(CuCl)]_2_ (Figure 1). The complex features a core structure of two Cu^I^ ions, bridged by two iodide ions. Each Cu^I^-ion is also coordinated by one equivalent of (*R*)-BINAP. Both cations show a distorted tetrahedral geometry being surrounded by two I atoms and two P atoms from the (*R*)-BINAP-ligands. The complex consists of isolated, butterfly-shaped molecules: The two planes defined by Cu(1), I(1) and I(2) respectively Cu(2), I(1) and I(2) form an angle of 146.11 (2)°. The structure contains disordered water. The absolute configuration of this chiral complex has been determined by anomalous dispersion effects. Four diordered water molecules are included in the unit cell, as shown in Figure 2: There are 2 voids in the crystal structure. This could be assigned as 2 water molecules per void or 4 water molecules per unit cell.

The structure displays a intramolecular C—H···I hydrogen bonding and contains disordered water, data regarding this is given in Table 1.

Table 2 compares selected distances and angles of the title compound of this study with the chloride-analog analyzed by Hattori and coworkers. The geometry is affected by the enlarged anions, resulting in a massively reduced Cu—*X*—Cu-angle for *X* = iodide. As a result of the rigid backbone of (*R*)-BINAP, neither the bonding distances of Cu—P, nor the angles P—Cu—P are disrupted.

### Experimental

The title compound has been synthesized unintentionally according to a modified protocol of Kunkely *et al.* (2008) by reaction of a modified N-donor-ligand with (*R*)-BINAP and copper iodide, changing the solvent from acetonitrile to dichloromethane at room temperature. The resulting suspension was filtered over a 45 µm disc-filter yielding a yellow solution. Crystals suitable for analysis were gained by slow diffusion of pentane in dichloromethane.

### Refinement

All H-atoms were geometrical positioned and refined using a riding model with fixed individual displacement parameters [U(H) = 1.2 *U*_eq_(C)] and with a C—H distance of 0.95 Å. The H atoms of the disordered water molecules could not be located and were omitted from refinement.

### Crystal data

**Table:**

[Cu_2_I_2_(C_44_H_32_P_2_)_2_]·0.67H_2_O	*D*_x_ = 1.550 Mg m^−^^3^
*M**_r_* = 1638.16	Mo *K*α radiation, λ = 0.71073 Å
Hexagonal, *P*6_3_	Cell parameters from 482 reflections
*a* = 25.573 (3) Å	θ = 2.5–25.0°
*c* = 18.593 (2) Å	µ = 1.63 mm^−^^1^
*V* = 10530 (2) Å^3^	*T* = 123 K
*Z* = 6	Blocks, yellow
*F*(000) = 4936	0.40 × 0.20 × 0.15 mm

### Data collection

**Table:**

Bruker–Nonius KappaCCD diffractometer	16098 independent reflections
Radiation source: fine-focus sealed tube	15085 reflections with *I* > 2σ(*I*)
Graphite monochromator	*R*_int_ = 0.041
rotation in φ and ω, 1 ° scans	θ_max_ = 27.5°, θ_min_ = 3.0°
Absorption correction: multi-scan (*SADABS*; Sheldrick, 1996)	*h* = −33→33
*T*_min_ = 0.696, *T*_max_ = 0.801	*k* = −33→33
187180 measured reflections	*l* = −24→24

### Refinement

**Table:**

Refinement on *F*^2^	Secondary atom site location: difference Fourier map
Least-squares matrix: full	Hydrogen site location: inferred from neighbouring sites
*R*[*F*^2^ > 2σ(*F*^2^)] = 0.030	H-atom parameters constrained
*wR*(*F*^2^) = 0.081	*w* = 1/[σ^2^(*F*_o_^2^) + (0.0381*P*)^2^ + 15.830*P*] where *P* = (*F*_o_^2^ + 2*F*_c_^2^)/3
*S* = 1.09	(Δ/σ)_max_ = 0.003
16098 reflections	Δρ_max_ = 1.62 e Å^−^^3^
873 parameters	Δρ_min_ = −0.57 e Å^−^^3^
1 restraint	Absolute structure: Flack (1983), 7802 Friedel pairs
Primary atom site location: structure-invariant direct methods	Flack parameter: −0.014 (9)

### Special details

**Table:**

Experimental. dx = 45 mm, 160 sec./°., 1 °., 9 sets, 859 frames
Geometry. All e.s.d.'s (except the e.s.d. in the dihedral angle between two l.s. planes) are estimated using the full covariance matrix. The cell e.s.d.'s are taken into account individually in the estimation of e.s.d.'s in distances, angles and torsion angles; correlations between e.s.d.'s in cell parameters are only used when they are defined by crystal symmetry. An approximate (isotropic) treatment of cell e.s.d.'s is used for estimating e.s.d.'s involving l.s. planes.
Refinement. Refinement of *F*^2^ against ALL reflections. The weighted *R*-factor *wR* and goodness of fit *S* are based on *F*^2^, conventional *R*-factors *R* are based on *F*, with *F* set to zero for negative *F*^2^. The threshold expression of *F*^2^ > σ(*F*^2^) is used only for calculating *R*-factors(gt) *etc*. and is not relevant to the choice of reflections for refinement. *R*-factors based on *F*^2^ are statistically about twice as large as those based on *F*, and *R*- factors based on ALL data will be even larger. 2 water molecules disordered about 2 position (s.o.f.= 1/3) and the 3-fold axis (Wyckoff letter a).Using SQUEEZE there are 2 voids in the crystal structure in 0,0,*z* and 0,0,*z* + 1/2 with 40 electrons. This could be assigned as 2 water molecules per void or 4 water molecules per unit cell. In the difference Fourier 2 peaks are found, which are refined as 1/3 water molecule, respectively (6 *x* 0.33333 water molecules = 2 water molecules). See also the SQUEEZE output included in the cif-file, even if the SQUEEZE-data are not used for the refinement.

### Fractional atomic coordinates and isotropic or equivalent isotropic displacement parameters (Å^2^)

**Table:**

	*x*	*y*	*z*	*U*_iso_*/*U*_eq_	Occ. (<1)
I1	0.649693 (11)	0.635358 (10)	0.530482 (12)	0.02538 (6)	
I2	0.639453 (10)	0.634461 (10)	0.752730 (11)	0.02365 (5)	
Cu1	0.587858 (17)	0.564088 (17)	0.63856 (3)	0.02186 (8)	
Cu2	0.664298 (19)	0.707200 (18)	0.64190 (3)	0.02543 (9)	
P1	0.60181 (4)	0.48336 (4)	0.65234 (5)	0.02005 (17)	
P2	0.48484 (4)	0.51442 (4)	0.62751 (5)	0.02196 (18)	
P3	0.61597 (4)	0.76126 (4)	0.65309 (5)	0.02046 (18)	
P4	0.76011 (4)	0.78585 (4)	0.62223 (5)	0.02411 (19)	
C1	0.53582 (15)	0.43087 (16)	0.70518 (18)	0.0185 (6)	
C2	0.54183 (17)	0.43578 (17)	0.78088 (19)	0.0233 (7)	
H2	0.5801	0.4627	0.8013	0.028*	
C3	0.49428 (18)	0.40297 (17)	0.82468 (19)	0.0240 (7)	
H3	0.4988	0.4090	0.8752	0.029*	
C4	0.43803 (18)	0.35992 (18)	0.7960 (2)	0.0241 (8)	
C5	0.38897 (19)	0.32306 (19)	0.8411 (2)	0.0297 (8)	
H5	0.3935	0.3276	0.8918	0.036*	
C6	0.3358 (2)	0.2816 (2)	0.8129 (2)	0.0352 (9)	
H6	0.3032	0.2567	0.8440	0.042*	
C7	0.32773 (17)	0.27438 (18)	0.7383 (2)	0.0299 (8)	
H7	0.2902	0.2442	0.7191	0.036*	
C8	0.37409 (17)	0.31097 (17)	0.6931 (2)	0.0253 (7)	
H8	0.3679	0.3068	0.6426	0.030*	
C9	0.43090 (17)	0.35477 (17)	0.72019 (19)	0.0225 (7)	
C10	0.48000 (16)	0.39400 (16)	0.67476 (18)	0.0188 (7)	
C11	0.46868 (15)	0.39819 (16)	0.59613 (18)	0.0188 (7)	
C12	0.45826 (15)	0.35021 (16)	0.54791 (18)	0.0202 (7)	
C13	0.45643 (16)	0.29703 (17)	0.5715 (2)	0.0255 (7)	
H13	0.4620	0.2924	0.6211	0.031*	
C14	0.44686 (17)	0.25196 (17)	0.5250 (2)	0.0301 (8)	
H14	0.4453	0.2162	0.5421	0.036*	
C15	0.43927 (19)	0.2588 (2)	0.4508 (2)	0.0348 (9)	
H15	0.4330	0.2276	0.4181	0.042*	
C16	0.44089 (18)	0.30915 (19)	0.4261 (2)	0.0313 (8)	
H16	0.4360	0.3132	0.3761	0.038*	
C17	0.44978 (16)	0.35621 (18)	0.4740 (2)	0.0249 (8)	
C18	0.44980 (17)	0.40842 (18)	0.44962 (18)	0.0262 (8)	
H18	0.4446	0.4128	0.3998	0.031*	
C19	0.45718 (17)	0.45264 (17)	0.49620 (19)	0.0254 (7)	
H19	0.4560	0.4869	0.4786	0.031*	
C20	0.46665 (15)	0.44849 (16)	0.57057 (19)	0.0206 (7)	
C21	0.60440 (15)	0.43927 (17)	0.57674 (19)	0.0226 (7)	
C22	0.6177 (2)	0.4643 (2)	0.5096 (2)	0.0352 (9)	
H22	0.6254	0.5043	0.5025	0.042*	
C23	0.6200 (2)	0.4309 (3)	0.4512 (2)	0.0448 (12)	
H23	0.6296	0.4484	0.4046	0.054*	
C24	0.6084 (2)	0.3732 (2)	0.4613 (2)	0.0408 (11)	
H24	0.6093	0.3503	0.4215	0.049*	
C25	0.59558 (17)	0.34817 (19)	0.5282 (3)	0.0355 (9)	
H25	0.5881	0.3082	0.5350	0.043*	
C26	0.59347 (16)	0.38085 (18)	0.5860 (2)	0.0285 (8)	
H26	0.5844	0.3632	0.6326	0.034*	
C27	0.66515 (17)	0.48976 (18)	0.7048 (2)	0.0255 (7)	
C28	0.71805 (17)	0.5444 (2)	0.7045 (2)	0.0304 (8)	
H28	0.7199	0.5781	0.6808	0.037*	
C29	0.76995 (18)	0.5502 (2)	0.7397 (2)	0.0386 (10)	
H29	0.8069	0.5876	0.7391	0.046*	
C30	0.7660 (2)	0.5013 (2)	0.7746 (2)	0.0419 (11)	
H30	0.8007	0.5052	0.7983	0.050*	
C31	0.7136 (2)	0.4469 (2)	0.7763 (3)	0.0417 (10)	
H31	0.7118	0.4135	0.8010	0.050*	
C32	0.66275 (18)	0.4411 (2)	0.7411 (2)	0.0338 (9)	
H32	0.6261	0.4034	0.7420	0.041*	
C33	0.43621 (16)	0.48265 (16)	0.7058 (2)	0.0234 (7)	
C34	0.45836 (17)	0.50764 (17)	0.7731 (2)	0.0262 (8)	
H34	0.4989	0.5396	0.7778	0.031*	
C35	0.42181 (19)	0.48635 (19)	0.8332 (2)	0.0310 (8)	
H35	0.4371	0.5038	0.8790	0.037*	
C36	0.36272 (19)	0.4395 (2)	0.8265 (2)	0.0338 (9)	
H36	0.3375	0.4247	0.8678	0.041*	
C37	0.34066 (17)	0.41437 (18)	0.7596 (2)	0.0316 (8)	
H37	0.3002	0.3823	0.7550	0.038*	
C38	0.37709 (17)	0.43557 (17)	0.6996 (2)	0.0267 (8)	
H38	0.3617	0.4179	0.6539	0.032*	
C39	0.45006 (17)	0.55435 (17)	0.5848 (2)	0.0258 (7)	
C40	0.48143 (19)	0.59543 (18)	0.5306 (2)	0.0327 (8)	
H40	0.5184	0.5997	0.5136	0.039*	
C41	0.4592 (2)	0.6308 (2)	0.5007 (3)	0.0395 (10)	
H41	0.4812	0.6588	0.4635	0.047*	
C42	0.4067 (2)	0.6252 (2)	0.5245 (3)	0.0418 (10)	
H42	0.3914	0.6486	0.5035	0.050*	
C43	0.3753 (2)	0.5855 (2)	0.5792 (3)	0.0419 (10)	
H43	0.3390	0.5825	0.5967	0.050*	
C44	0.3965 (2)	0.5498 (2)	0.6091 (2)	0.0343 (9)	
H44	0.3742	0.5220	0.6463	0.041*	
C45	0.67026 (16)	0.83028 (16)	0.70100 (18)	0.0206 (7)	
C46	0.66571 (18)	0.83087 (18)	0.7775 (2)	0.0270 (8)	
H46	0.6325	0.7980	0.8008	0.032*	
C47	0.70765 (19)	0.87730 (19)	0.8177 (2)	0.0293 (8)	
H47	0.7040	0.8762	0.8686	0.035*	
C48	0.75699 (17)	0.92745 (17)	0.7837 (2)	0.0253 (7)	
C49	0.80224 (19)	0.9761 (2)	0.8245 (2)	0.0334 (9)	
H49	0.7999	0.9755	0.8755	0.040*	
C50	0.84912 (19)	1.02371 (19)	0.7901 (3)	0.0361 (10)	
H50	0.8789	1.0563	0.8177	0.043*	
C51	0.85406 (18)	1.02537 (19)	0.7159 (3)	0.0346 (9)	
H51	0.8866	1.0592	0.6930	0.042*	
C52	0.81193 (17)	0.97827 (17)	0.6756 (2)	0.0271 (8)	
H52	0.8158	0.9794	0.6247	0.033*	
C53	0.76274 (17)	0.92790 (17)	0.7087 (2)	0.0235 (7)	
C54	0.71932 (15)	0.87694 (15)	0.66794 (18)	0.0182 (6)	
C55	0.72983 (16)	0.87513 (16)	0.58945 (19)	0.0220 (7)	
C56	0.71696 (16)	0.91078 (16)	0.54075 (19)	0.0238 (7)	
C57	0.69856 (16)	0.95133 (17)	0.5653 (2)	0.0249 (7)	
H57	0.6956	0.9564	0.6155	0.030*	
C58	0.68494 (18)	0.98328 (17)	0.5177 (2)	0.0296 (8)	
H58	0.6729	1.0106	0.5351	0.036*	
C59	0.68847 (19)	0.97635 (18)	0.4432 (2)	0.0316 (8)	
H59	0.6790	0.9990	0.4106	0.038*	
C60	0.70551 (18)	0.93721 (19)	0.4180 (2)	0.0310 (8)	
H60	0.7074	0.9323	0.3676	0.037*	
C61	0.72060 (18)	0.90340 (18)	0.4664 (2)	0.0274 (8)	
C62	0.7374 (2)	0.8621 (2)	0.4412 (2)	0.0330 (9)	
H62	0.7392	0.8566	0.3909	0.040*	
C63	0.75106 (19)	0.8299 (2)	0.4882 (2)	0.0318 (9)	
H63	0.7621	0.8021	0.4700	0.038*	
C64	0.74921 (17)	0.83683 (18)	0.5631 (2)	0.0256 (8)	
C65	0.54671 (16)	0.73259 (17)	0.70608 (19)	0.0234 (7)	
C66	0.51108 (17)	0.67069 (18)	0.7147 (2)	0.0281 (8)	
H66	0.5250	0.6447	0.6978	0.034*	
C67	0.45519 (17)	0.64653 (18)	0.7477 (2)	0.0335 (8)	
H67	0.4308	0.6040	0.7529	0.040*	
C68	0.43473 (18)	0.68321 (19)	0.7728 (2)	0.0330 (9)	
H68	0.3958	0.6662	0.7941	0.040*	
C69	0.4709 (2)	0.74537 (19)	0.7673 (2)	0.0336 (9)	
H69	0.4575	0.7711	0.7864	0.040*	
C70	0.52652 (19)	0.76983 (18)	0.7339 (2)	0.0317 (9)	
H70	0.5512	0.8124	0.7300	0.038*	
C71	0.59484 (17)	0.78534 (16)	0.5708 (2)	0.0243 (7)	
C72	0.57366 (19)	0.82556 (19)	0.5709 (2)	0.0306 (8)	
H72	0.5699	0.8420	0.6151	0.037*	
C73	0.5579 (2)	0.8420 (2)	0.5071 (2)	0.0349 (9)	
H73	0.5443	0.8705	0.5075	0.042*	
C74	0.5617 (2)	0.81693 (19)	0.4427 (2)	0.0342 (9)	
H74	0.5500	0.8273	0.3989	0.041*	
C75	0.5827 (2)	0.77695 (19)	0.4428 (2)	0.0355 (9)	
H75	0.5859	0.7600	0.3987	0.043*	
C76	0.59936 (19)	0.76105 (18)	0.5067 (2)	0.0300 (8)	
H76	0.6139	0.7334	0.5060	0.036*	
C77	0.81622 (19)	0.7730 (2)	0.5766 (2)	0.0331 (9)	
C78	0.8623 (2)	0.8159 (2)	0.5340 (3)	0.0451 (11)	
H78	0.8633	0.8526	0.5225	0.054*	
C79	0.9070 (3)	0.8050 (3)	0.5080 (3)	0.0619 (16)	
H79	0.9385	0.8345	0.4789	0.074*	
C80	0.9062 (3)	0.7533 (4)	0.5236 (4)	0.072 (2)	
H80	0.9374	0.7467	0.5061	0.086*	
C81	0.8609 (3)	0.7105 (3)	0.5644 (4)	0.0647 (18)	
H81	0.8601	0.6737	0.5743	0.078*	
C82	0.8154 (2)	0.7200 (2)	0.5920 (3)	0.0468 (12)	
H82	0.7842	0.6901	0.6210	0.056*	
C83	0.80673 (18)	0.83112 (18)	0.6976 (2)	0.0277 (8)	
C84	0.7904 (2)	0.80764 (18)	0.7662 (2)	0.0352 (9)	
H84	0.7546	0.7700	0.7736	0.042*	
C85	0.8268 (3)	0.8394 (2)	0.8243 (3)	0.0452 (12)	
H85	0.8155	0.8233	0.8714	0.054*	
C86	0.8786 (2)	0.8938 (2)	0.8144 (3)	0.0463 (12)	
H86	0.9029	0.9155	0.8544	0.056*	
C87	0.89505 (18)	0.9166 (2)	0.7461 (3)	0.0421 (10)	
H87	0.9312	0.9539	0.7390	0.050*	
C88	0.85970 (18)	0.8859 (2)	0.6876 (2)	0.0331 (9)	
H88	0.8716	0.9021	0.6406	0.040*	
O1W	0.9617 (5)	1.0055 (5)	0.5573 (6)	0.051 (3)*	0.33
O2W	0.9552 (6)	0.9714 (7)	0.4700 (8)	0.073 (4)*	0.33

### Atomic displacement parameters (Å^2^)

**Table:**

	*U*^11^	*U*^22^	*U*^33^	*U*^12^	*U*^13^	*U*^23^
I1	0.03306 (12)	0.02501 (11)	0.01841 (11)	0.01478 (10)	−0.00013 (10)	−0.00215 (10)
I2	0.02630 (11)	0.02620 (11)	0.01602 (10)	0.01130 (9)	−0.00312 (9)	−0.00279 (9)
Cu1	0.02024 (18)	0.02337 (19)	0.02140 (18)	0.01048 (15)	−0.00347 (18)	−0.00214 (19)
Cu2	0.0336 (2)	0.02417 (19)	0.02182 (18)	0.01691 (18)	−0.0005 (2)	−0.0017 (2)
P1	0.0178 (4)	0.0251 (4)	0.0184 (4)	0.0116 (3)	−0.0038 (3)	−0.0039 (3)
P2	0.0194 (4)	0.0235 (4)	0.0246 (5)	0.0120 (3)	−0.0048 (3)	−0.0018 (3)
P3	0.0219 (4)	0.0192 (4)	0.0195 (5)	0.0097 (3)	−0.0004 (3)	−0.0032 (3)
P4	0.0265 (4)	0.0266 (4)	0.0236 (5)	0.0166 (4)	−0.0006 (3)	−0.0030 (3)
C1	0.0208 (16)	0.0232 (16)	0.0174 (15)	0.0155 (14)	−0.0018 (12)	−0.0021 (12)
C2	0.0258 (17)	0.0279 (18)	0.0212 (16)	0.0171 (15)	−0.0071 (13)	−0.0043 (14)
C3	0.036 (2)	0.0310 (19)	0.0171 (16)	0.0261 (17)	−0.0051 (14)	−0.0045 (14)
C4	0.033 (2)	0.0298 (19)	0.0214 (17)	0.0243 (17)	0.0031 (14)	0.0024 (14)
C5	0.040 (2)	0.037 (2)	0.0197 (17)	0.0254 (19)	0.0068 (16)	0.0057 (15)
C6	0.037 (2)	0.038 (2)	0.034 (2)	0.0216 (19)	0.0128 (17)	0.0121 (18)
C7	0.0227 (17)	0.0304 (19)	0.034 (2)	0.0114 (15)	0.0015 (15)	0.0056 (15)
C8	0.0240 (18)	0.0294 (19)	0.0244 (17)	0.0149 (16)	−0.0005 (14)	0.0017 (14)
C9	0.0251 (18)	0.0256 (18)	0.0228 (17)	0.0172 (15)	0.0004 (14)	−0.0008 (14)
C10	0.0203 (16)	0.0226 (16)	0.0184 (16)	0.0144 (14)	−0.0023 (12)	−0.0036 (13)
C11	0.0135 (15)	0.0240 (17)	0.0177 (15)	0.0084 (13)	−0.0010 (12)	−0.0014 (13)
C12	0.0160 (15)	0.0244 (17)	0.0177 (16)	0.0081 (13)	0.0011 (12)	0.0004 (12)
C13	0.0243 (18)	0.0325 (19)	0.0218 (17)	0.0157 (16)	−0.0030 (14)	−0.0037 (14)
C14	0.0313 (19)	0.0264 (17)	0.033 (2)	0.0148 (15)	−0.0024 (17)	−0.0049 (16)
C15	0.035 (2)	0.037 (2)	0.030 (2)	0.0156 (18)	−0.0058 (17)	−0.0171 (17)
C16	0.030 (2)	0.035 (2)	0.0202 (17)	0.0097 (17)	−0.0019 (14)	−0.0091 (15)
C17	0.0172 (16)	0.0320 (19)	0.0195 (17)	0.0079 (15)	−0.0028 (13)	−0.0037 (14)
C18	0.0264 (18)	0.0331 (19)	0.0133 (15)	0.0107 (16)	−0.0017 (13)	0.0008 (14)
C19	0.0228 (17)	0.0282 (19)	0.0217 (17)	0.0100 (15)	−0.0041 (13)	0.0020 (14)
C20	0.0154 (15)	0.0229 (16)	0.0208 (16)	0.0076 (13)	−0.0058 (12)	−0.0039 (13)
C21	0.0168 (16)	0.0337 (19)	0.0237 (17)	0.0173 (15)	−0.0071 (13)	−0.0110 (14)
C22	0.047 (2)	0.049 (2)	0.0264 (19)	0.037 (2)	−0.0019 (17)	−0.0069 (17)
C23	0.070 (3)	0.076 (3)	0.0167 (18)	0.057 (3)	−0.0030 (19)	−0.005 (2)
C24	0.048 (3)	0.063 (3)	0.032 (2)	0.043 (3)	−0.0164 (19)	−0.023 (2)
C25	0.0279 (19)	0.039 (2)	0.046 (2)	0.0216 (17)	−0.0080 (19)	−0.015 (2)
C26	0.0192 (17)	0.0300 (19)	0.038 (2)	0.0138 (15)	−0.0017 (15)	−0.0050 (16)
C27	0.0222 (17)	0.037 (2)	0.0220 (17)	0.0181 (16)	−0.0029 (14)	−0.0067 (15)
C28	0.0218 (18)	0.039 (2)	0.030 (2)	0.0141 (17)	−0.0001 (15)	−0.0077 (16)
C29	0.0184 (17)	0.052 (3)	0.037 (2)	0.0121 (18)	0.0003 (16)	−0.015 (2)
C30	0.031 (2)	0.069 (3)	0.037 (2)	0.033 (2)	−0.0105 (18)	−0.016 (2)
C31	0.036 (2)	0.060 (3)	0.042 (2)	0.034 (2)	−0.0089 (19)	−0.007 (2)
C32	0.0280 (19)	0.039 (2)	0.041 (2)	0.0221 (17)	−0.0088 (17)	−0.0050 (18)
C33	0.0234 (17)	0.0246 (17)	0.0284 (19)	0.0167 (15)	−0.0007 (14)	−0.0013 (14)
C34	0.0262 (18)	0.0252 (17)	0.0304 (19)	0.0153 (15)	−0.0041 (14)	−0.0065 (14)
C35	0.034 (2)	0.039 (2)	0.029 (2)	0.0254 (18)	−0.0021 (16)	−0.0059 (16)
C36	0.034 (2)	0.044 (2)	0.036 (2)	0.029 (2)	0.0117 (17)	0.0076 (18)
C37	0.0248 (17)	0.0329 (19)	0.044 (2)	0.0195 (16)	0.0018 (17)	0.0041 (18)
C38	0.0245 (18)	0.0287 (19)	0.0312 (19)	0.0165 (16)	−0.0033 (15)	−0.0025 (15)
C39	0.0247 (18)	0.0249 (18)	0.0272 (18)	0.0119 (15)	−0.0090 (14)	−0.0009 (14)
C40	0.039 (2)	0.039 (2)	0.0257 (18)	0.0238 (18)	−0.0016 (18)	0.0038 (18)
C41	0.048 (3)	0.042 (2)	0.035 (2)	0.028 (2)	−0.0051 (19)	0.0098 (19)
C42	0.049 (3)	0.039 (2)	0.050 (3)	0.031 (2)	−0.007 (2)	0.002 (2)
C43	0.042 (3)	0.045 (3)	0.051 (3)	0.032 (2)	−0.003 (2)	0.002 (2)
C44	0.035 (2)	0.034 (2)	0.042 (2)	0.0234 (18)	−0.0024 (18)	−0.0005 (17)
C45	0.0253 (17)	0.0226 (17)	0.0175 (16)	0.0147 (14)	−0.0016 (13)	−0.0030 (13)
C46	0.0308 (19)	0.0293 (19)	0.0208 (17)	0.0150 (16)	0.0015 (14)	−0.0004 (14)
C47	0.041 (2)	0.036 (2)	0.0162 (16)	0.0235 (19)	−0.0024 (15)	−0.0038 (15)
C48	0.0273 (18)	0.0234 (17)	0.0301 (18)	0.0163 (15)	−0.0057 (15)	−0.0054 (14)
C49	0.036 (2)	0.036 (2)	0.035 (2)	0.0232 (19)	−0.0115 (17)	−0.0134 (17)
C50	0.026 (2)	0.030 (2)	0.050 (3)	0.0126 (17)	−0.0142 (18)	−0.0200 (19)
C51	0.0211 (19)	0.027 (2)	0.051 (3)	0.0089 (16)	−0.0045 (17)	−0.0088 (18)
C52	0.0247 (18)	0.0278 (19)	0.0303 (19)	0.0142 (16)	−0.0016 (15)	−0.0018 (15)
C53	0.0236 (17)	0.0229 (17)	0.0264 (18)	0.0134 (15)	−0.0019 (14)	−0.0030 (14)
C54	0.0197 (16)	0.0188 (15)	0.0183 (15)	0.0113 (13)	−0.0013 (12)	−0.0031 (12)
C55	0.0199 (16)	0.0217 (17)	0.0217 (18)	0.0084 (14)	0.0007 (13)	0.0002 (13)
C56	0.0240 (16)	0.0225 (16)	0.0216 (17)	0.0091 (14)	0.0014 (13)	0.0019 (13)
C57	0.0253 (18)	0.0250 (18)	0.0232 (17)	0.0117 (15)	−0.0021 (14)	0.0015 (14)
C58	0.035 (2)	0.0280 (18)	0.028 (2)	0.0175 (16)	−0.0032 (15)	0.0018 (15)
C59	0.038 (2)	0.0287 (19)	0.0259 (19)	0.0147 (17)	−0.0045 (16)	0.0071 (15)
C60	0.030 (2)	0.036 (2)	0.0220 (18)	0.0132 (17)	0.0022 (14)	0.0063 (15)
C61	0.0280 (19)	0.0297 (19)	0.0228 (18)	0.0132 (16)	0.0040 (14)	0.0038 (15)
C62	0.040 (2)	0.046 (2)	0.0166 (17)	0.024 (2)	0.0059 (16)	0.0013 (16)
C63	0.036 (2)	0.044 (2)	0.0261 (19)	0.0282 (19)	0.0056 (16)	0.0006 (16)
C64	0.0255 (18)	0.0312 (19)	0.0231 (17)	0.0163 (16)	0.0020 (14)	−0.0035 (15)
C65	0.0235 (17)	0.0257 (18)	0.0196 (16)	0.0113 (15)	0.0015 (13)	−0.0020 (13)
C66	0.0253 (18)	0.0268 (19)	0.033 (2)	0.0139 (16)	−0.0033 (15)	−0.0011 (15)
C67	0.0229 (17)	0.0297 (19)	0.039 (2)	0.0067 (15)	−0.0014 (17)	0.0018 (18)
C68	0.0279 (19)	0.039 (2)	0.031 (2)	0.0161 (17)	0.0058 (15)	0.0025 (16)
C69	0.040 (2)	0.038 (2)	0.031 (2)	0.0263 (19)	0.0052 (17)	−0.0010 (16)
C70	0.035 (2)	0.0247 (18)	0.033 (2)	0.0135 (17)	0.0052 (16)	0.0001 (15)
C71	0.0252 (18)	0.0238 (17)	0.0243 (17)	0.0126 (15)	−0.0040 (14)	−0.0014 (14)
C72	0.035 (2)	0.033 (2)	0.0263 (19)	0.0196 (18)	0.0011 (16)	−0.0017 (16)
C73	0.040 (2)	0.039 (2)	0.035 (2)	0.026 (2)	−0.0037 (17)	−0.0021 (17)
C74	0.041 (2)	0.036 (2)	0.0276 (19)	0.0206 (19)	−0.0063 (17)	0.0009 (17)
C75	0.055 (3)	0.034 (2)	0.0223 (19)	0.026 (2)	−0.0052 (18)	−0.0074 (16)
C76	0.038 (2)	0.0293 (19)	0.0273 (18)	0.0201 (17)	−0.0027 (16)	−0.0038 (15)
C77	0.035 (2)	0.040 (2)	0.032 (2)	0.0255 (19)	−0.0069 (17)	−0.0118 (17)
C78	0.042 (2)	0.066 (3)	0.040 (2)	0.037 (2)	0.006 (2)	0.000 (2)
C79	0.053 (3)	0.100 (5)	0.046 (3)	0.049 (4)	0.011 (2)	−0.006 (3)
C80	0.071 (4)	0.125 (6)	0.062 (4)	0.081 (4)	−0.014 (3)	−0.036 (4)
C81	0.071 (4)	0.068 (4)	0.087 (4)	0.058 (4)	−0.030 (4)	−0.037 (3)
C82	0.043 (3)	0.042 (3)	0.067 (3)	0.030 (2)	−0.019 (2)	−0.018 (2)
C83	0.0289 (19)	0.0287 (19)	0.0306 (19)	0.0183 (16)	−0.0051 (15)	−0.0076 (15)
C84	0.047 (2)	0.0275 (19)	0.035 (2)	0.0215 (18)	−0.0108 (18)	−0.0072 (16)
C85	0.068 (3)	0.046 (3)	0.033 (2)	0.037 (3)	−0.016 (2)	−0.012 (2)
C86	0.045 (3)	0.047 (3)	0.057 (3)	0.030 (2)	−0.022 (2)	−0.023 (2)
C87	0.0225 (18)	0.038 (2)	0.064 (3)	0.0134 (17)	−0.012 (2)	−0.015 (2)
C88	0.0246 (19)	0.038 (2)	0.037 (2)	0.0166 (17)	0.0005 (16)	−0.0059 (17)

### Geometric parameters (Å, º)

**Table:**

I1—Cu1	2.6416 (6)	C40—H40	0.9500
I1—Cu2	2.6684 (6)	C41—C42	1.353 (7)
I2—Cu2	2.6321 (6)	C41—H41	0.9500
I2—Cu1	2.6667 (6)	C42—C43	1.376 (7)
Cu1—P1	2.2786 (10)	C42—H42	0.9500
Cu1—P2	2.2913 (10)	C43—C44	1.387 (6)
Cu2—P3	2.2781 (10)	C43—H43	0.9500
Cu2—P4	2.2923 (11)	C44—H44	0.9500
P1—C21	1.824 (4)	C45—C54	1.371 (5)
P1—C27	1.826 (4)	C45—C46	1.428 (5)
P1—C1	1.830 (4)	C46—C47	1.359 (6)
P2—C33	1.820 (4)	C46—H46	0.9500
P2—C39	1.836 (4)	C47—C48	1.421 (6)
P2—C20	1.843 (4)	C47—H47	0.9500
P3—C71	1.829 (4)	C48—C53	1.402 (5)
P3—C65	1.829 (4)	C48—C49	1.421 (5)
P3—C45	1.840 (4)	C49—C50	1.367 (7)
P4—C83	1.829 (4)	C49—H49	0.9500
P4—C64	1.830 (4)	C50—C51	1.385 (6)
P4—C77	1.832 (4)	C50—H50	0.9500
C1—C10	1.379 (5)	C51—C52	1.370 (6)
C1—C2	1.415 (5)	C51—H51	0.9500
C2—C3	1.351 (5)	C52—C53	1.415 (5)
C2—H2	0.9500	C52—H52	0.9500
C3—C4	1.408 (6)	C53—C54	1.435 (5)
C3—H3	0.9500	C54—C55	1.489 (5)
C4—C5	1.409 (5)	C55—C64	1.390 (5)
C4—C9	1.418 (5)	C55—C56	1.435 (5)
C5—C6	1.345 (6)	C56—C61	1.405 (5)
C5—H5	0.9500	C56—C57	1.412 (5)
C6—C7	1.401 (6)	C57—C58	1.363 (5)
C6—H6	0.9500	C57—H57	0.9500
C7—C8	1.370 (5)	C58—C59	1.405 (6)
C7—H7	0.9500	C58—H58	0.9500
C8—C9	1.411 (5)	C59—C60	1.359 (6)
C8—H8	0.9500	C59—H59	0.9500
C9—C10	1.427 (5)	C60—C61	1.429 (6)
C10—C11	1.504 (5)	C60—H60	0.9500
C11—C20	1.396 (5)	C61—C62	1.404 (6)
C11—C12	1.433 (5)	C62—C63	1.360 (6)
C12—C13	1.407 (5)	C62—H62	0.9500
C12—C17	1.411 (5)	C63—C64	1.408 (5)
C13—C14	1.362 (5)	C63—H63	0.9500
C13—H13	0.9500	C65—C66	1.385 (5)
C14—C15	1.415 (6)	C65—C70	1.390 (5)
C14—H14	0.9500	C66—C67	1.385 (6)
C15—C16	1.349 (6)	C66—H66	0.9500
C15—H15	0.9500	C67—C68	1.365 (6)
C16—C17	1.422 (5)	C67—H67	0.9500
C16—H16	0.9500	C68—C69	1.387 (6)
C17—C18	1.410 (6)	C68—H68	0.9500
C18—C19	1.360 (5)	C69—C70	1.382 (6)
C18—H18	0.9500	C69—H69	0.9500
C19—C20	1.417 (5)	C70—H70	0.9500
C19—H19	0.9500	C71—C76	1.376 (5)
C21—C22	1.366 (6)	C71—C72	1.381 (5)
C21—C26	1.387 (5)	C72—C73	1.386 (6)
C22—C23	1.401 (6)	C72—H72	0.9500
C22—H22	0.9500	C73—C74	1.385 (6)
C23—C24	1.365 (7)	C73—H73	0.9500
C23—H23	0.9500	C74—C75	1.371 (6)
C24—C25	1.360 (7)	C74—H74	0.9500
C24—H24	0.9500	C75—C76	1.389 (6)
C25—C26	1.379 (6)	C75—H75	0.9500
C25—H25	0.9500	C76—H76	0.9500
C26—H26	0.9500	C77—C82	1.375 (6)
C27—C28	1.375 (6)	C77—C78	1.388 (7)
C27—C32	1.391 (6)	C78—C79	1.391 (7)
C28—C29	1.419 (6)	C78—H78	0.9500
C28—H28	0.9500	C79—C80	1.343 (10)
C29—C30	1.368 (7)	C79—H79	0.9500
C29—H29	0.9500	C80—C81	1.360 (10)
C30—C31	1.368 (7)	C80—H80	0.9500
C30—H30	0.9500	C81—C82	1.400 (7)
C31—C32	1.395 (5)	C81—H81	0.9500
C31—H31	0.9500	C82—H82	0.9500
C32—H32	0.9500	C83—C84	1.382 (6)
C33—C38	1.388 (5)	C83—C88	1.390 (6)
C33—C34	1.391 (5)	C84—C85	1.391 (6)
C34—C35	1.382 (6)	C84—H84	0.9500
C34—H34	0.9500	C85—C86	1.373 (8)
C35—C36	1.387 (6)	C85—H85	0.9500
C35—H35	0.9500	C86—C87	1.371 (8)
C36—C37	1.384 (6)	C86—H86	0.9500
C36—H36	0.9500	C87—C88	1.382 (6)
C37—C38	1.380 (6)	C87—H87	0.9500
C37—H37	0.9500	C88—H88	0.9500
C38—H38	0.9500	O1W—O2W	1.810 (18)
C39—C40	1.386 (6)	O2W—O2W^i^	1.74 (2)
C39—C44	1.391 (6)	O2W—O2W^ii^	1.74 (2)
C40—C41	1.402 (6)		
			
Cu1—I1—Cu2	73.374 (17)	C40—C39—P2	118.6 (3)
Cu2—I2—Cu1	73.554 (17)	C44—C39—P2	123.0 (3)
P1—Cu1—P2	99.52 (3)	C39—C40—C41	120.7 (4)
P1—Cu1—I1	113.56 (3)	C39—C40—H40	119.6
P2—Cu1—I1	116.10 (3)	C41—C40—H40	119.6
P1—Cu1—I2	105.85 (3)	C42—C41—C40	120.1 (4)
P2—Cu1—I2	119.29 (3)	C42—C41—H41	120.0
I1—Cu1—I2	102.499 (17)	C40—C41—H41	120.0
P3—Cu2—P4	98.53 (3)	C41—C42—C43	120.2 (4)
P3—Cu2—I2	110.17 (3)	C41—C42—H42	119.9
P4—Cu2—I2	121.29 (3)	C43—C42—H42	119.9
P3—Cu2—I1	123.85 (3)	C42—C43—C44	120.4 (4)
P4—Cu2—I1	101.56 (3)	C42—C43—H43	119.8
I2—Cu2—I1	102.708 (17)	C44—C43—H43	119.8
C21—P1—C27	99.34 (16)	C43—C44—C39	120.5 (4)
C21—P1—C1	105.39 (17)	C43—C44—H44	119.8
C27—P1—C1	103.32 (17)	C39—C44—H44	119.8
C21—P1—Cu1	122.96 (13)	C54—C45—C46	118.8 (3)
C27—P1—Cu1	120.82 (13)	C54—C45—P3	122.8 (3)
C1—P1—Cu1	102.72 (11)	C46—C45—P3	117.8 (3)
C33—P2—C39	100.42 (17)	C47—C46—C45	121.7 (4)
C33—P2—C20	104.31 (17)	C47—C46—H46	119.2
C39—P2—C20	106.85 (16)	C45—C46—H46	119.2
C33—P2—Cu1	121.03 (12)	C46—C47—C48	120.1 (4)
C39—P2—Cu1	118.22 (13)	C46—C47—H47	120.0
C20—P2—Cu1	104.65 (11)	C48—C47—H47	120.0
C71—P3—C65	101.28 (17)	C53—C48—C47	119.5 (4)
C71—P3—C45	106.87 (16)	C53—C48—C49	119.1 (4)
C65—P3—C45	103.81 (16)	C47—C48—C49	121.3 (4)
C71—P3—Cu2	117.98 (12)	C50—C49—C48	119.9 (4)
C65—P3—Cu2	121.08 (12)	C50—C49—H49	120.0
C45—P3—Cu2	104.37 (11)	C48—C49—H49	120.0
C83—P4—C64	107.02 (18)	C49—C50—C51	121.2 (4)
C83—P4—C77	98.44 (18)	C49—C50—H50	119.4
C64—P4—C77	105.15 (19)	C51—C50—H50	119.4
C83—P4—Cu2	120.37 (14)	C52—C51—C50	119.9 (4)
C64—P4—Cu2	104.13 (13)	C52—C51—H51	120.0
C77—P4—Cu2	120.42 (15)	C50—C51—H51	120.0
C10—C1—C2	119.9 (3)	C51—C52—C53	120.8 (4)
C10—C1—P1	122.8 (3)	C51—C52—H52	119.6
C2—C1—P1	116.7 (3)	C53—C52—H52	119.6
C3—C2—C1	121.4 (4)	C48—C53—C52	118.9 (4)
C3—C2—H2	119.3	C48—C53—C54	119.2 (3)
C1—C2—H2	119.3	C52—C53—C54	121.9 (3)
C2—C3—C4	120.5 (3)	C45—C54—C53	120.6 (3)
C2—C3—H3	119.7	C45—C54—C55	120.9 (3)
C4—C3—H3	119.7	C53—C54—C55	118.5 (3)
C3—C4—C5	121.1 (3)	C64—C55—C56	120.1 (3)
C3—C4—C9	118.9 (4)	C64—C55—C54	120.1 (3)
C5—C4—C9	120.0 (4)	C56—C55—C54	119.8 (3)
C6—C5—C4	120.5 (4)	C61—C56—C57	119.1 (3)
C6—C5—H5	119.8	C61—C56—C55	118.9 (3)
C4—C5—H5	119.8	C57—C56—C55	121.9 (3)
C5—C6—C7	120.9 (4)	C58—C57—C56	120.6 (4)
C5—C6—H6	119.5	C58—C57—H57	119.7
C7—C6—H6	119.5	C56—C57—H57	119.7
C8—C7—C6	119.8 (4)	C57—C58—C59	120.9 (4)
C8—C7—H7	120.1	C57—C58—H58	119.6
C6—C7—H7	120.1	C59—C58—H58	119.6
C7—C8—C9	121.3 (4)	C60—C59—C58	119.9 (4)
C7—C8—H8	119.4	C60—C59—H59	120.1
C9—C8—H8	119.4	C58—C59—H59	120.1
C8—C9—C4	117.5 (4)	C59—C60—C61	120.7 (4)
C8—C9—C10	122.8 (3)	C59—C60—H60	119.6
C4—C9—C10	119.7 (4)	C61—C60—H60	119.6
C1—C10—C9	119.0 (3)	C62—C61—C56	119.7 (4)
C1—C10—C11	121.4 (3)	C62—C61—C60	121.4 (4)
C9—C10—C11	119.5 (3)	C56—C61—C60	118.9 (4)
C20—C11—C12	119.9 (3)	C63—C62—C61	120.6 (4)
C20—C11—C10	119.6 (3)	C63—C62—H62	119.7
C12—C11—C10	120.5 (3)	C61—C62—H62	119.7
C13—C12—C17	118.4 (3)	C62—C63—C64	121.6 (4)
C13—C12—C11	122.3 (3)	C62—C63—H63	119.2
C17—C12—C11	119.3 (3)	C64—C63—H63	119.2
C14—C13—C12	121.7 (4)	C55—C64—C63	118.9 (4)
C14—C13—H13	119.2	C55—C64—P4	121.7 (3)
C12—C13—H13	119.2	C63—C64—P4	118.6 (3)
C13—C14—C15	119.5 (4)	C66—C65—C70	118.8 (4)
C13—C14—H14	120.3	C66—C65—P3	118.1 (3)
C15—C14—H14	120.3	C70—C65—P3	122.9 (3)
C16—C15—C14	120.6 (4)	C67—C66—C65	120.2 (4)
C16—C15—H15	119.7	C67—C66—H66	119.9
C14—C15—H15	119.7	C65—C66—H66	119.9
C15—C16—C17	120.7 (4)	C68—C67—C66	120.7 (4)
C15—C16—H16	119.7	C68—C67—H67	119.7
C17—C16—H16	119.7	C66—C67—H67	119.7
C18—C17—C12	119.3 (3)	C67—C68—C69	119.8 (4)
C18—C17—C16	121.6 (3)	C67—C68—H68	120.1
C12—C17—C16	119.1 (4)	C69—C68—H68	120.1
C19—C18—C17	121.1 (3)	C70—C69—C68	119.8 (4)
C19—C18—H18	119.5	C70—C69—H69	120.1
C17—C18—H18	119.4	C68—C69—H69	120.1
C18—C19—C20	121.1 (4)	C69—C70—C65	120.5 (4)
C18—C19—H19	119.4	C69—C70—H70	119.7
C20—C19—H19	119.4	C65—C70—H70	119.7
C11—C20—C19	119.2 (3)	C76—C71—C72	119.4 (4)
C11—C20—P2	122.6 (3)	C76—C71—P3	117.9 (3)
C19—C20—P2	117.8 (3)	C72—C71—P3	122.7 (3)
C22—C21—C26	119.2 (4)	C71—C72—C73	120.4 (4)
C22—C21—P1	119.5 (3)	C71—C72—H72	119.8
C26—C21—P1	121.4 (3)	C73—C72—H72	119.8
C21—C22—C23	119.9 (4)	C74—C73—C72	120.1 (4)
C21—C22—H22	120.1	C74—C73—H73	120.0
C23—C22—H22	120.1	C72—C73—H73	120.0
C24—C23—C22	120.0 (4)	C75—C74—C73	119.3 (4)
C24—C23—H23	120.0	C75—C74—H74	120.3
C22—C23—H23	120.0	C73—C74—H74	120.3
C25—C24—C23	120.3 (4)	C74—C75—C76	120.7 (4)
C25—C24—H24	119.8	C74—C75—H75	119.6
C23—C24—H24	119.8	C76—C75—H75	119.6
C24—C25—C26	120.0 (4)	C71—C76—C75	120.1 (4)
C24—C25—H25	120.0	C71—C76—H76	120.0
C26—C25—H25	120.0	C75—C76—H76	120.0
C25—C26—C21	120.6 (4)	C82—C77—C78	119.1 (4)
C25—C26—H26	119.7	C82—C77—P4	116.9 (4)
C21—C26—H26	119.7	C78—C77—P4	123.7 (3)
C28—C27—C32	119.4 (4)	C77—C78—C79	119.8 (5)
C28—C27—P1	117.9 (3)	C77—C78—H78	120.1
C32—C27—P1	122.6 (3)	C79—C78—H78	120.1
C27—C28—C29	119.9 (4)	C80—C79—C78	120.9 (6)
C27—C28—H28	120.0	C80—C79—H79	119.6
C29—C28—H28	120.0	C78—C79—H79	119.6
C30—C29—C28	119.1 (4)	C79—C80—C81	120.0 (5)
C30—C29—H29	120.5	C79—C80—H80	120.0
C28—C29—H29	120.5	C81—C80—H80	120.0
C29—C30—C31	121.8 (4)	C80—C81—C82	120.8 (6)
C29—C30—H30	119.1	C80—C81—H81	119.6
C31—C30—H30	119.1	C82—C81—H81	119.6
C30—C31—C32	119.1 (5)	C77—C82—C81	119.4 (6)
C30—C31—H31	120.5	C77—C82—H82	120.3
C32—C31—H31	120.5	C81—C82—H82	120.3
C27—C32—C31	120.7 (4)	C84—C83—C88	119.4 (4)
C27—C32—H32	119.6	C84—C83—P4	118.2 (3)
C31—C32—H32	119.6	C88—C83—P4	122.2 (3)
C38—C33—C34	119.3 (4)	C83—C84—C85	119.6 (4)
C38—C33—P2	121.7 (3)	C83—C84—H84	120.2
C34—C33—P2	118.9 (3)	C85—C84—H84	120.2
C35—C34—C33	120.5 (4)	C86—C85—C84	120.9 (5)
C35—C34—H34	119.8	C86—C85—H85	119.6
C33—C34—H34	119.8	C84—C85—H85	119.6
C34—C35—C36	119.9 (4)	C87—C86—C85	119.3 (4)
C34—C35—H35	120.1	C87—C86—H86	120.3
C36—C35—H35	120.1	C85—C86—H86	120.3
C37—C36—C35	119.7 (4)	C86—C87—C88	120.9 (4)
C37—C36—H36	120.1	C86—C87—H87	119.6
C35—C36—H36	120.1	C88—C87—H87	119.6
C38—C37—C36	120.4 (4)	C87—C88—C83	119.9 (4)
C38—C37—H37	119.8	C87—C88—H88	120.1
C36—C37—H37	119.8	C83—C88—H88	120.1
C37—C38—C33	120.2 (4)	O2W^i^—O2W—O2W^ii^	60.000 (7)
C37—C38—H38	119.9	O2W^i^—O2W—O1W	94.7 (6)
C33—C38—H38	119.9	O2W^ii^—O2W—O1W	70.3 (7)
C40—C39—C44	118.1 (4)		
			
Cu2—I1—Cu1—P1	−134.86 (3)	C20—P2—C33—C34	142.9 (3)
Cu2—I1—Cu1—P2	110.65 (3)	Cu1—P2—C33—C34	25.6 (3)
Cu2—I1—Cu1—I2	−21.158 (14)	C38—C33—C34—C35	−0.7 (5)
Cu2—I2—Cu1—P1	140.69 (3)	P2—C33—C34—C35	177.2 (3)
Cu2—I2—Cu1—P2	−108.43 (3)	C33—C34—C35—C36	0.4 (6)
Cu2—I2—Cu1—I1	21.443 (15)	C34—C35—C36—C37	−0.1 (6)
Cu1—I2—Cu2—P3	112.49 (3)	C35—C36—C37—C38	0.1 (6)
Cu1—I2—Cu2—P4	−133.42 (3)	C36—C37—C38—C33	−0.4 (6)
Cu1—I2—Cu2—I1	−21.236 (15)	C34—C33—C38—C37	0.7 (5)
Cu1—I1—Cu2—P3	−103.77 (4)	P2—C33—C38—C37	−177.1 (3)
Cu1—I1—Cu2—P4	147.60 (3)	C33—P2—C39—C40	166.7 (3)
Cu1—I1—Cu2—I2	21.468 (15)	C20—P2—C39—C40	−84.7 (3)
P2—Cu1—P1—C21	82.03 (14)	Cu1—P2—C39—C40	32.8 (4)
I1—Cu1—P1—C21	−42.01 (14)	C33—P2—C39—C44	−6.8 (4)
I2—Cu1—P1—C21	−153.68 (14)	C20—P2—C39—C44	101.8 (4)
P2—Cu1—P1—C27	−150.24 (14)	Cu1—P2—C39—C44	−140.7 (3)
I1—Cu1—P1—C27	85.72 (15)	C44—C39—C40—C41	−0.7 (6)
I2—Cu1—P1—C27	−25.95 (15)	P2—C39—C40—C41	−174.5 (3)
P2—Cu1—P1—C1	−36.05 (12)	C39—C40—C41—C42	0.0 (7)
I1—Cu1—P1—C1	−160.09 (11)	C40—C41—C42—C43	1.2 (7)
I2—Cu1—P1—C1	88.24 (11)	C41—C42—C43—C44	−1.7 (8)
P1—Cu1—P2—C33	76.45 (14)	C42—C43—C44—C39	1.0 (7)
I1—Cu1—P2—C33	−161.30 (13)	C40—C39—C44—C43	0.2 (6)
I2—Cu1—P2—C33	−37.86 (14)	P2—C39—C44—C43	173.7 (3)
P1—Cu1—P2—C39	−159.34 (15)	C71—P3—C45—C54	−48.9 (3)
I1—Cu1—P2—C39	−37.09 (15)	C65—P3—C45—C54	−155.4 (3)
I2—Cu1—P2—C39	86.35 (15)	Cu2—P3—C45—C54	76.9 (3)
P1—Cu1—P2—C20	−40.64 (12)	C71—P3—C45—C46	140.7 (3)
I1—Cu1—P2—C20	81.60 (12)	C65—P3—C45—C46	34.1 (3)
I2—Cu1—P2—C20	−154.95 (12)	Cu2—P3—C45—C46	−93.6 (3)
P4—Cu2—P3—C71	81.28 (14)	C54—C45—C46—C47	2.2 (6)
I2—Cu2—P3—C71	−150.79 (14)	P3—C45—C46—C47	173.0 (3)
I1—Cu2—P3—C71	−28.88 (15)	C45—C46—C47—C48	1.2 (6)
P4—Cu2—P3—C65	−153.34 (14)	C46—C47—C48—C53	−1.5 (6)
I2—Cu2—P3—C65	−25.42 (14)	C46—C47—C48—C49	−178.6 (4)
I1—Cu2—P3—C65	96.50 (14)	C53—C48—C49—C50	2.9 (6)
P4—Cu2—P3—C45	−37.10 (12)	C47—C48—C49—C50	−179.9 (4)
I2—Cu2—P3—C45	90.83 (12)	C48—C49—C50—C51	−0.9 (6)
I1—Cu2—P3—C45	−147.26 (12)	C49—C50—C51—C52	−1.0 (7)
P3—Cu2—P4—C83	79.91 (15)	C50—C51—C52—C53	0.7 (6)
I2—Cu2—P4—C83	−40.04 (15)	C47—C48—C53—C52	179.7 (3)
I1—Cu2—P4—C83	−152.82 (14)	C49—C48—C53—C52	−3.1 (6)
P3—Cu2—P4—C64	−39.91 (12)	C47—C48—C53—C54	−1.4 (6)
I2—Cu2—P4—C64	−159.86 (12)	C49—C48—C53—C54	175.8 (3)
I1—Cu2—P4—C64	87.36 (12)	C51—C52—C53—C48	1.3 (6)
P3—Cu2—P4—C77	−157.32 (16)	C51—C52—C53—C54	−177.5 (4)
I2—Cu2—P4—C77	82.73 (16)	C46—C45—C54—C53	−5.1 (5)
I1—Cu2—P4—C77	−30.04 (16)	P3—C45—C54—C53	−175.5 (3)
C21—P1—C1—C10	−48.5 (3)	C46—C45—C54—C55	173.0 (3)
C27—P1—C1—C10	−152.3 (3)	P3—C45—C54—C55	2.7 (5)
Cu1—P1—C1—C10	81.4 (3)	C48—C53—C54—C45	4.8 (5)
C21—P1—C1—C2	140.7 (3)	C52—C53—C54—C45	−176.3 (3)
C27—P1—C1—C2	36.9 (3)	C48—C53—C54—C55	−173.4 (3)
Cu1—P1—C1—C2	−89.5 (3)	C52—C53—C54—C55	5.5 (5)
C10—C1—C2—C3	2.9 (5)	C45—C54—C55—C64	−72.9 (5)
P1—C1—C2—C3	174.0 (3)	C53—C54—C55—C64	105.3 (4)
C1—C2—C3—C4	4.1 (5)	C45—C54—C55—C56	104.0 (4)
C2—C3—C4—C5	176.5 (3)	C53—C54—C55—C56	−77.8 (4)
C2—C3—C4—C9	−4.6 (5)	C64—C55—C56—C61	4.0 (5)
C3—C4—C5—C6	−179.0 (4)	C54—C55—C56—C61	−172.9 (3)
C9—C4—C5—C6	2.1 (6)	C64—C55—C56—C57	−178.5 (3)
C4—C5—C6—C7	−0.6 (6)	C54—C55—C56—C57	4.7 (5)
C5—C6—C7—C8	−1.5 (6)	C61—C56—C57—C58	−0.5 (6)
C6—C7—C8—C9	2.1 (6)	C55—C56—C57—C58	−178.1 (4)
C7—C8—C9—C4	−0.6 (6)	C56—C57—C58—C59	0.5 (6)
C7—C8—C9—C10	−179.4 (4)	C57—C58—C59—C60	0.2 (6)
C3—C4—C9—C8	179.7 (3)	C58—C59—C60—C61	−0.7 (6)
C5—C4—C9—C8	−1.5 (6)	C57—C56—C61—C62	−178.4 (4)
C3—C4—C9—C10	−1.5 (5)	C55—C56—C61—C62	−0.8 (6)
C5—C4—C9—C10	177.3 (3)	C57—C56—C61—C60	0.0 (5)
C2—C1—C10—C9	−9.0 (5)	C55—C56—C61—C60	177.6 (3)
P1—C1—C10—C9	−179.5 (3)	C59—C60—C61—C62	179.0 (4)
C2—C1—C10—C11	165.7 (3)	C59—C60—C61—C56	0.6 (6)
P1—C1—C10—C11	−4.8 (5)	C56—C61—C62—C63	−1.0 (6)
C8—C9—C10—C1	−173.0 (3)	C60—C61—C62—C63	−179.4 (4)
C4—C9—C10—C1	8.3 (5)	C61—C62—C63—C64	−0.3 (7)
C8—C9—C10—C11	12.3 (5)	C56—C55—C64—C63	−5.3 (6)
C4—C9—C10—C11	−166.5 (3)	C54—C55—C64—C63	171.6 (4)
C1—C10—C11—C20	−70.8 (4)	C56—C55—C64—P4	−174.8 (3)
C9—C10—C11—C20	103.9 (4)	C54—C55—C64—P4	2.1 (5)
C1—C10—C11—C12	111.0 (4)	C62—C63—C64—C55	3.5 (7)
C9—C10—C11—C12	−74.3 (4)	C62—C63—C64—P4	173.3 (3)
C20—C11—C12—C13	−176.4 (3)	C83—P4—C64—C55	−50.2 (4)
C10—C11—C12—C13	1.8 (5)	C77—P4—C64—C55	−154.2 (3)
C20—C11—C12—C17	3.9 (5)	Cu2—P4—C64—C55	78.3 (3)
C10—C11—C12—C17	−177.9 (3)	C83—P4—C64—C63	140.2 (3)
C17—C12—C13—C14	0.1 (5)	C77—P4—C64—C63	36.2 (4)
C11—C12—C13—C14	−179.6 (3)	Cu2—P4—C64—C63	−91.3 (3)
C12—C13—C14—C15	0.8 (6)	C71—P3—C65—C66	107.0 (3)
C13—C14—C15—C16	−0.6 (6)	C45—P3—C65—C66	−142.3 (3)
C14—C15—C16—C17	−0.5 (6)	Cu2—P3—C65—C66	−25.8 (3)
C13—C12—C17—C18	178.3 (3)	C71—P3—C65—C70	−68.7 (4)
C11—C12—C17—C18	−2.0 (5)	C45—P3—C65—C70	42.0 (4)
C13—C12—C17—C16	−1.2 (5)	Cu2—P3—C65—C70	158.5 (3)
C11—C12—C17—C16	178.5 (3)	C70—C65—C66—C67	2.8 (6)
C15—C16—C17—C18	−178.0 (4)	P3—C65—C66—C67	−173.1 (3)
C15—C16—C17—C12	1.4 (6)	C65—C66—C67—C68	−0.7 (6)
C12—C17—C18—C19	−0.7 (6)	C66—C67—C68—C69	−1.9 (7)
C16—C17—C18—C19	178.7 (4)	C67—C68—C69—C70	2.4 (6)
C17—C18—C19—C20	1.6 (6)	C68—C69—C70—C65	−0.3 (6)
C12—C11—C20—C19	−3.1 (5)	C66—C65—C70—C69	−2.3 (6)
C10—C11—C20—C19	178.7 (3)	P3—C65—C70—C69	173.4 (3)
C12—C11—C20—P2	−175.5 (3)	C65—P3—C71—C76	−123.4 (3)
C10—C11—C20—P2	6.3 (5)	C45—P3—C71—C76	128.2 (3)
C18—C19—C20—C11	0.3 (5)	Cu2—P3—C71—C76	11.2 (4)
C18—C19—C20—P2	173.1 (3)	C65—P3—C71—C72	55.3 (4)
C33—P2—C20—C11	−53.6 (3)	C45—P3—C71—C72	−53.1 (4)
C39—P2—C20—C11	−159.4 (3)	Cu2—P3—C71—C72	−170.1 (3)
Cu1—P2—C20—C11	74.5 (3)	C76—C71—C72—C73	−0.9 (6)
C33—P2—C20—C19	133.9 (3)	P3—C71—C72—C73	−179.5 (3)
C39—P2—C20—C19	28.1 (3)	C71—C72—C73—C74	1.7 (7)
Cu1—P2—C20—C19	−98.1 (3)	C72—C73—C74—C75	−1.6 (7)
C27—P1—C21—C22	−115.1 (3)	C73—C74—C75—C76	0.7 (7)
C1—P1—C21—C22	138.2 (3)	C72—C71—C76—C75	0.0 (6)
Cu1—P1—C21—C22	21.4 (4)	P3—C71—C76—C75	178.7 (3)
C27—P1—C21—C26	64.5 (3)	C74—C75—C76—C71	0.1 (7)
C1—P1—C21—C26	−42.2 (3)	C83—P4—C77—C82	95.4 (4)
Cu1—P1—C21—C26	−158.9 (2)	C64—P4—C77—C82	−154.3 (3)
C26—C21—C22—C23	0.1 (6)	Cu2—P4—C77—C82	−37.4 (4)
P1—C21—C22—C23	179.8 (3)	C83—P4—C77—C78	−78.1 (4)
C21—C22—C23—C24	0.6 (7)	C64—P4—C77—C78	32.2 (4)
C22—C23—C24—C25	−1.1 (7)	Cu2—P4—C77—C78	149.1 (3)
C23—C24—C25—C26	0.8 (6)	C82—C77—C78—C79	−0.7 (7)
C24—C25—C26—C21	−0.1 (6)	P4—C77—C78—C79	172.7 (4)
C22—C21—C26—C25	−0.4 (5)	C77—C78—C79—C80	0.1 (9)
P1—C21—C26—C25	180.0 (3)	C78—C79—C80—C81	0.9 (9)
C21—P1—C27—C28	105.1 (3)	C79—C80—C81—C82	−1.4 (9)
C1—P1—C27—C28	−146.5 (3)	C78—C77—C82—C81	0.2 (7)
Cu1—P1—C27—C28	−32.6 (3)	P4—C77—C82—C81	−173.6 (4)
C21—P1—C27—C32	−71.2 (4)	C80—C81—C82—C77	0.9 (8)
C1—P1—C27—C32	37.1 (4)	C64—P4—C83—C84	135.9 (3)
Cu1—P1—C27—C32	151.0 (3)	C77—P4—C83—C84	−115.4 (3)
C32—C27—C28—C29	1.5 (6)	Cu2—P4—C83—C84	17.5 (4)
P1—C27—C28—C29	−175.0 (3)	C64—P4—C83—C88	−48.6 (4)
C27—C28—C29—C30	−1.2 (6)	C77—P4—C83—C88	60.2 (4)
C28—C29—C30—C31	0.3 (6)	Cu2—P4—C83—C88	−167.0 (3)
C29—C30—C31—C32	0.3 (7)	C88—C83—C84—C85	0.8 (6)
C28—C27—C32—C31	−1.0 (6)	P4—C83—C84—C85	176.4 (3)
P1—C27—C32—C31	175.4 (3)	C83—C84—C85—C86	0.0 (7)
C30—C31—C32—C27	0.1 (7)	C84—C85—C86—C87	−0.9 (7)
C39—P2—C33—C38	71.3 (3)	C85—C86—C87—C88	0.9 (7)
C20—P2—C33—C38	−39.3 (3)	C86—C87—C88—C83	−0.1 (6)
Cu1—P2—C33—C38	−156.5 (3)	C84—C83—C88—C87	−0.8 (6)
C39—P2—C33—C34	−106.5 (3)	P4—C83—C88—C87	−176.3 (3)

Symmetry codes: (i) −*y*+2, *x*−*y*+1, *z*; (ii) −*x*+*y*+1, −*x*+2, *z*.

### Hydrogen-bond geometry (Å, º)

**Table:**

*D*—H···*A*	*D*—H	H···*A*	*D*···*A*	*D*—H···*A*
C40—H40···I1	0.95	3.02	3.894 (4)	153

### Comparison of selected bond distances and angles (Å, °) for two (*R*)-BINAP–Cu–halide complexes.

**Table:**

Halide *X*	Cu—*X*	Cu—P	*X*—Cu—*X*	*X*—Cu—P	P—Cu—P	Cu—*X*—Cu
Iodide	2.641	2.28	102.5	113.6	99.5	73.3
Chloride	2.378	2.260	98.0	114.9	100.2	81.3

Values for the iodide complex are from this work, while data for the chloride complex were taken from Hattori *et al.* (2010).
